# Supplementary material for: Pre-mitotic genome re-organisation bookends the B cell differentiation process
Source: Nat Commun. 2021 Feb 26;12:1344. doi: 10.1038/s41467-021-21536-2 (PMC7910489; doi:10.1038/s41467-021-21536-2)
Supplement: Supplementary file 1 — Supplementary Information [file 41467_2021_21536_MOESM1_ESM.pdf]

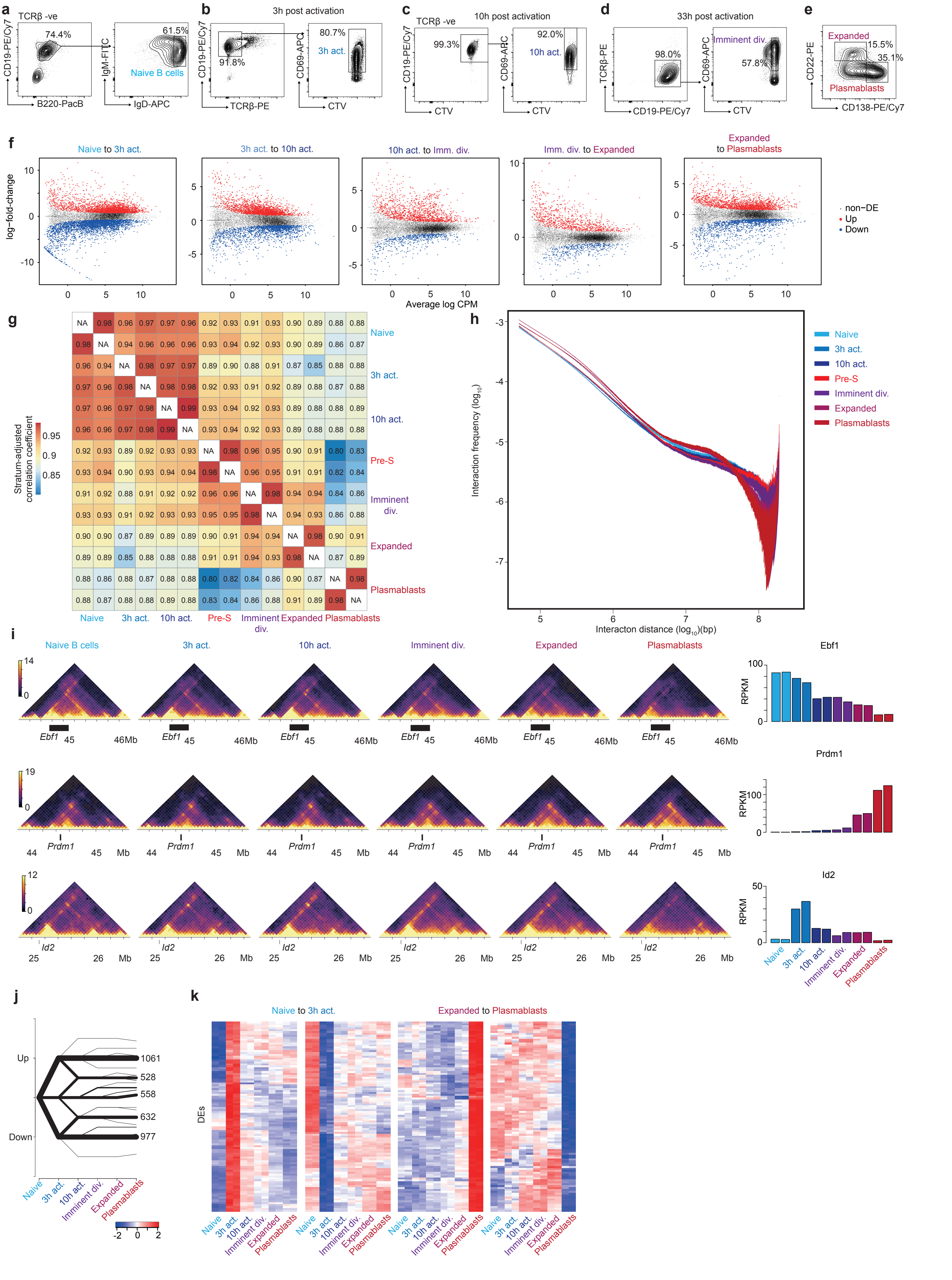

Supplementary Figure 1. Cell isolation, in situ HiC quality checks and expression and organisational changes across B cell differentiation a, Flow cytometry plots showing strategy used to isolate naïve B cells from mouse spleen. b-d, Flow cytometry plots showing strategy used to isolate activated B cells from culture 3 (b), 10 (c) and 33 hours (d) after their activation with lipopolysaccharide (LPS) using surface markers and Cell Trace Violet (CTV). e, Flow cytometry plot showing strategy used to isolate the expanded B cell population and plasmablasts from culture 4 days after their activation with lipopolysaccharide (LPS). f, Mean-difference (MD) plots with differentially expressed (DE) genes highlighted in red (up) and blue (down) for each transition of B cell activation. DE genes have fold-changes significantly greater than 1.5 (Treat FDR < 0.05). Plotted on the y-axis is log-intensity ratios of the genes and plotted on the x-axis is the log-intensity averages. g, Heatmap of the median reproducibility score (stratum adjusted correlation coefficient) across all chromosomes between replicate libraries at 50 kbp. h, Decay curves of summed libraries of the read-pair interaction frequency (log10) as a function of the interaction distance (log10). i, Normalized in-situ HiC contact matrices at 50 kbp showing genome organisation at the Ebf1 locus (chr4:44.2-45.5Mbp), Prdm1 locus (chr10:43.8-45.5Mbp) and the Id2 locus (chr12: 24.8-26.5Mbp) during all stages of B cell activation, with corresponding expression changes in reads per million per kilobase (RPKM) (far right). j, Plot showing the patterns and numbers within each pattern of DEs during B cell activation. k, Heatmap of logRPKMs of the top 100 differentially expressed genes (DEs) by false discovery rate in four select patterns expression change during B cell activation. The left panels show DEs that transiently increase or decrease in expression 3 hours post-activation before returning to their original level. The right panels show DEs that upregulate or downregulate exclusively during plasmablast differentiation.

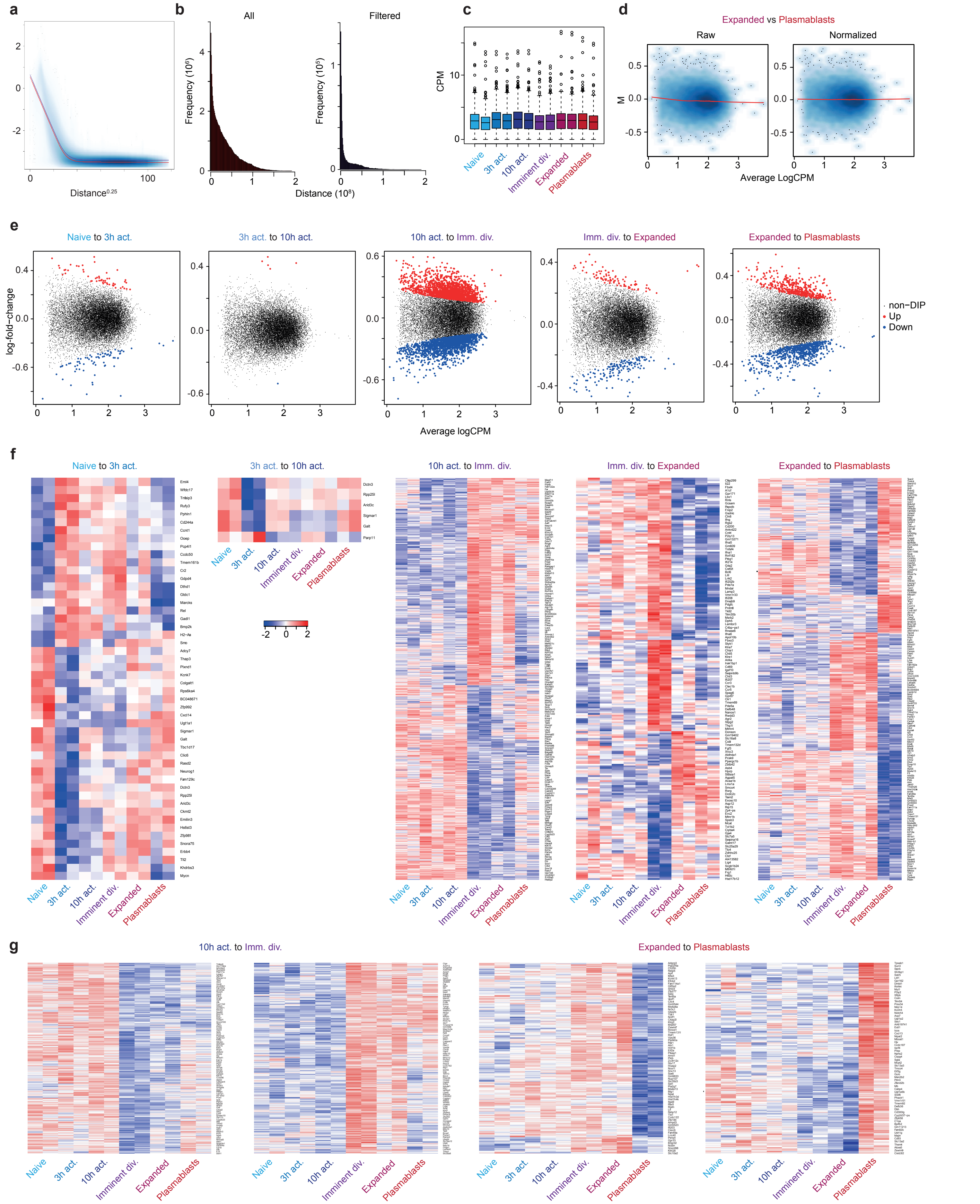

Supplementary Figure 2. Differential interactions at promoters a, A plot of the average log counts per million (logCPM) of interactions for all libraries as a function of interaction distance to the power of 0.25. Interaction distance is the number of bp between the boundaries of an interaction. A loess curve was fitted to the data with a span of 0.05 shown in red. For an interaction to be retained it was required to exceed the fitted curved plus two times the mean of the absolute values of the residuals from the loess fit shown in purple. b, Histograms of the interaction distance of all interactions before and after applying filtering. c, 1-99th percentile boxplots of the counts per million (CPM) of the aggregated promoter counts for each library. d, Mean-abundance (MA) plot of a naïve B library versus a plasmablast library before and after normalization. Plotted on the y-axis is the log-fold change of the interacting promoter counts between libraries and plotted on the x-axis is the log-intensity averages. e, Mean-difference (MD) plots with differentially interacting promoters (DIPs) highlighted in red (up) and blue (down) for each transition of B cell activation. f, Heatmap of logCPMs of all DIPs at each transition of B cell activation. g, Heatmap of logCPMs of all DIPs in patterns of DIPs that exclusively increase or decrease at the 10 hours activation to imminent division transition and expanded to plasmablasts transition.

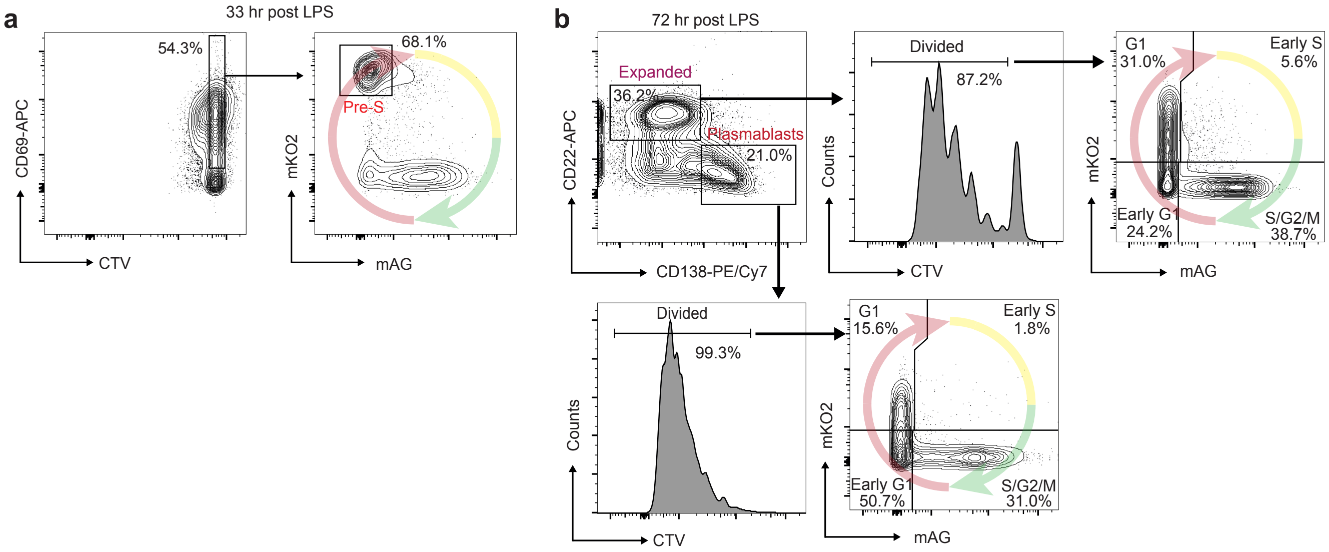

Supplementary Figure 3. Isolation Pre-S population and quantitation of 72-hour post-activation cell cycle from FUCCI mouse cell culture. a, Flow cytometry plots showing strategy for isolating undivided, activated, G1 cells 33 hours after lipopolysaccharide (LPS) exposure using Cell Trace Violet (CTV) dilution and monomeric Kusabira Orange 2 (mKO2) and monomeric Azami Green (mAG) expression. b, Flow cytometry plots showing strategy for determining the cell cycle states of expanded B cells and plasmablasts in 3 day cultures, as in Supp Fig 3a.
