# Supplementary material for: Pre-mitotic genome re-organisation bookends the B cell differentiation process
Source: Nat Commun. 2021 Feb 26;12:1344. doi: 10.1038/s41467-021-21536-2 (PMC7910489; doi:10.1038/s41467-021-21536-2)
Supplement: Supplementary file 3 — Description of Additional Supplementary Files [file 41467_2021_21536_MOESM3_ESM.pdf]

Description of Additional Supplementary Files

Supplementary Data 1
In situ HiC library statistics

Supplementary Data 2
Differentially expressed genes across B cell differentiation

Supplementary Data 3
Differential interactions across B cell differentiation

Supplementary Data 4
Pre-existing loop structure and gene expression changes three hours after activation

Supplementary Data 5
Gene ontology of differentially expressed genes across B cell differentiation

Supplementary Data 6
A/B compartment changes across B cell differentiation

Supplementary Data 7
Differential interactions at promoters (DIPs) across B cell differentiation

Supplementary Data 8
Patterns of change in differential interactions at promoters (DIPs) across B cell differentiation

Supplementary Data 9
Gene ontology of DIPs across B cell differentiation

Supplementary Data 10
Gene ontology of DIP patterns across B cell differentiation

Supplementary Data 11
Motif analysis within differentially interacting promoters

Supplementary Data 12
Differential interactions across B cell differentiation, including Pre-S phase population

Supplementary Data 13
Antibodies used in the study
