# Supplementary material for: Pre-mitotic genome re-organisation bookends the B cell differentiation process
Source: Nat Commun. 2021 Feb 26;12:1344. doi: 10.1038/s41467-021-21536-2 (PMC7910489; doi:10.1038/s41467-021-21536-2)
Supplement: Supplementary file 17 — Reporting Summary [file 41467_2021_21536_MOESM17_ESM.pdf]

nature research

Corresponding author(s): Rhys Allan

Last updated by author(s): Dec 14, 2020

Reporting Summary

Nature Research wishes to improve the reproducibility of the work that we publish. This form provides structure for consistency and transparency in reporting. For further information on Nature Research policies, see Authors & Referees and the Editorial Policy Checklist .

Statistics

For all statistical analyses, confirm that the following items are present in the figure legend, table legend, main text, or Methods section.

n/a	Confirmed	
☐	☒	The exact sample size (*n*) for each experimental group/condition, given as a discrete number and unit of measurement
☐	☒	A statement on whether measurements were taken from distinct samples or whether the same sample was measured repeatedly
☐	☒	The statistical test(s) used AND whether they are one- or two-sided<br>*Only common tests should be described solely by name; describe more complex techniques in the Methods section.*
☐	☒	A description of all covariates tested
☐	☒	A description of any assumptions or corrections, such as tests of normality and adjustment for multiple comparisons
☐	☒	A full description of the statistical parameters including central tendency (e.g. means) or other basic estimates (e.g. regression coefficient) AND variation (e.g. standard deviation) or associated estimates of uncertainty (e.g. confidence intervals)
☐	☒	For null hypothesis testing, the test statistic (e.g. *F*, *t*, *r*) with confidence intervals, effect sizes, degrees of freedom and *P* value noted<br>*Give P values as exact values whenever suitable.*
☐	☒	For Bayesian analysis, information on the choice of priors and Markov chain Monte Carlo settings
☐	☒	For hierarchical and complex designs, identification of the appropriate level for tests and full reporting of outcomes
☐	☒	Estimates of effect sizes (e.g. Cohen's *d*, Pearson's *r*), indicating how they were calculated

Our web collection on statistics for biologists contains articles on many of the points above.

Software and code

Policy information about availability of computer code

Data collection	Standard Illumina NextSeq 500 procedures and software. Base calling and quality scoring were determined using Real-Time Analysis on board.<br>BD Aria III, BD LSRFortessa
Data analysis	In no particular order: diffHic v1.16.0 (Lun and Smyth, 2015), bowtie2 v2.2.5 (Langmead and Salzberg, 2012), Picard suite v1.117 (https://broadinstitute.github.io/picard/) , quasi-likelihood (QL) framework (Lund et al., 2012) of the edgeR package v3.26.5, csaw package v1.18.0 (Lun and Smyth, 2016), TADbit v0.2.0.5 python based software (Serra et al., 2016), limma package v3.40.2 (Ritchie et al., 2015), Sushi R package v1.22.0 (Phanstiel, 2015), rtracklayer package v1.32.2 (Lawrence et al., 2009), Rsubread v1.28.0 (Liao et al., 2013), MACS2 v2.1.0 (Zhang et al., 2008), HOMER v4.11 (Heinz et al., 2010), MEME v5.0.5 (McLeay and Bailey, 2010), cutadapt v0.9.5 (Martin, 2011), HiCRep v1.14.0 (Yang et al. 2017), viridisLite v0.3.0 (Garnier, 2018), IRanges v2.20.2 (Lawrence et al. 2013)<br>FlowJo 10.4.1

For manuscripts utilizing custom algorithms or software that are central to the research but not yet described in published literature, software must be made available to editors/reviewers. We strongly encourage code deposition in a community repository (e.g. GitHub). See the Nature Research guidelines for submitting code & software for further information.

Data

Policy information about availability of data

All manuscripts must include a data availability statement. This statement should provide the following information, where applicable:

- Accession codes, unique identifiers, or web links for publicly available datasets
- A list of figures that have associated raw data
- A description of any restrictions on data availability

Sequence data that support the findings of this study are tabulated in the supplementary tables and are available in the GEO database under accession numbers GSE147497 and GSE99151.

Relative motif enrichment was performed with the program ame of the MEME v5.0.5 89 software package on each pattern. The motif database used was HOCOMOCOv11_full_MOUSE from MEME.

Field-specific reporting

Please select the one below that is the best fit for your research. If you are not sure, read the appropriate sections before making your selection.

- [x] Life sciences
- [ ] Behavioural & social sciences
- [ ] Ecological, evolutionary & environmental sciences

For a reference copy of the document with all sections, see nature.com/documents/nr-reporting-summary-flat.pdf

Life sciences study design

All studies must disclose on these points even when the disclosure is negative.

Sample size	Sequence experiments were performed in duplicate. From previous previous experience, and as demonstrated in Figures 1b, 1g and Supplementary Figure 1g, the Hi-C and RNA-seq experiments are highly reproducible with duplicate libraries showing minimal variation. These were sufficient to determine differences between samples.<br>In mouse experiments, data was generated from 4 independent experiments. The reproducible experimental data suggests this sample size is sufficient and allows rigorous statistical testing. Sample size is stated in each figure legend.
Data exclusions	No exclusions
Replication	Hi-C and RNA-seq have been replicated twice, whereas mouse experimental data have been obtained from 4 independent experiments.
Randomization	All males were randomly chosen from the relevant pool.
Blinding	Results were analysed without blinding of grouping.<br>Analysis is all computational, no blinding required.

Reporting for specific materials, systems and methods

We require information from authors about some types of materials, experimental systems and methods used in many studies. Here, indicate whether each material, system or method listed is relevant to your study. If you are not sure if a list item applies to your research, read the appropriate section before selecting a response.

Materials & experimental systems

n/a	Involved in the study	
[ ]	[x]	Antibodies
[x]	[ ]	Eukaryotic cell lines
[x]	[ ]	Palaeontology
[ ]	[x]	Animals and other organisms
[x]	[ ]	Human research participants
[x]	[ ]	Clinical data

Methods

n/a	Involved in the study	
[x]	[ ]	ChIP-seq
[ ]	[x]	Flow cytometry
[x]	[ ]	MRI-based neuroimaging

Antibodies

Antibodies used	Target-Fluorophore-Clone-Vendor<br>TCRβ-PE-H57-597-BD 562841<br>CD19-PE/Cy7-ID3-BD 561739<br>B220-PacB-RA3-6B2-WEHI in house<br>IgM-FITC-X-54-Miltenyi 130-095-906<br>IgD-APC-11-26c.2a-Miltenyi 130-102-445<br>CD69-APC-H1.2F3-Miltenyi 130-103-980<br>CD138-PE/Cy7-281-2-Biolegend 142513<br>CD22-PE-OX-97-Biolegend 126111<br>CD22-APC-OX-97-Biolegend 126109<br>Dilutions used for each Ab have been indicated in Supplementary Table 13.
Validation	Antibody validations were performed by antibody suppliers per quality assurance literature provided by each supplier, and were used as guidelines. Also the antibodies were validated and titrated by using positive/negative control cells from C57BL/6 mouse before the experiments.

Animals and other organisms

Policy information about studies involving animals; ARRIVE guidelines recommended for reporting animal research

Laboratory animals	Experiments were performed using male C57BL/6, FUCCI (Sakaue-Sawano et al, 2008) or Blimp1-transgenic mice (Kallies et al, 2004) at age 6-12 w. Mice were housed in a specific pathogen-free environment with ambient temperature around 23˚C, humidity 40-60% and light/dark cycle of 12h/12h.
Wild animals	No wild animals were used in this study.
Field-collected samples	No field-collected samples were used in this study.
Ethics oversight	Walter and Eliza Hall Institute Animal Ethics Committee (#2016-003)

Note that full information on the approval of the study protocol must also be provided in the manuscript.

Flow Cytometry

Plots

Confirm that:

- [x] The axis labels state the marker and fluorochrome used (e.g. CD4-FITC).
- [x] The axis scales are clearly visible. Include numbers along axes only for bottom left plot of group (a 'group' is an analysis of identical markers).
- [x] All plots are contour plots with outliers or pseudocolor plots.
- [x] A numerical value for number of cells or percentage (with statistics) is provided.

Methodology

Sample preparation	Splenocytes were obtained from mechanically homogenized organs
Instrument	Flow cytometric analyses were performed on BD FACSCanto or BD LSRFortessa with sorting performed on the BD FACS Aria III.
Software	FlowJo v10.4.1
Cell population abundance	Purity was checked and always exceeded 97%
Gating strategy	All cells were initially gated on FSC-A/SSC-A, followed by doublet exclusion by FSC-H/FSC-A, then dead cells were gated out by using PI as viability dye. For naive B cells, they were first gated as B220-PB and CD19-PE/Cy7, then on IgM-FITC and IgD-APC. For 3h, 10h and 33h activated B cells, they were first gated on TCRβ-PE and CD19-PE/Cy7, then on CD69-APC and CTV. For Expanded activated B cells and plasmablasts, the live cells were gated straight on CD22-PE and CD138-PE/Cy7.<br>For FUCCI cell cycle analyses, cells were first gated on CD22-APC and CD138-PE/Cy7, then on CTV, followed by mKO2 and mAG.<br>For Blimp1 mice analyses, live cells were gated on Blimp1-GFP vs SSC-A.<br>All the gating strategies are shown in Data figures

- [x] Tick this box to confirm that a figure exemplifying the gating strategy is provided in the Supplementary Information.
